# Presentation to a Hospital Engineer

**Published:** 1919-12-06

**Authors:** 


### Presentation to a Hospital Engineer.

Mr. Charles H. Horner, whose health has led him to retire after twenty-five years' service as engineer to the Derbyshire Royal Infirmary, has been presented by the permanent staff with two easy chairs. The presentation was made by Mr. Walter Banks, the Superintendent, and Mrs. Horner was also included in the presentation.

